# A Nomogram Integrating Clinical and Ultrasonographic Features for Preoperative Differentiation of Invasive Ductal Carcinoma and Invasive Lobular Carcinoma of the Breast

**DOI:** 10.3390/diagnostics16132008

**Published:** 2026-06-27

**Authors:** Deqing Zhang, Yuqing Zhang, Yan Li

**Affiliations:** 1 Department of Medical Imaging, The Second Qilu Hospital of Shandong University, Jinan 250033, China; 18764043776@163.com; 2 Department of Ultrasound, The Second Qilu Hospital of Shandong University, Jinan 250033, China; 3 Department of Nuclear Medicine, The Second Qilu Hospital of Shandong University, Jinan 250033, China; liyan-0633@163.com

**Keywords:** breast, invasive ductal carcinoma, invasive lobular carcinoma, nomogram, ultrasound, clinical features

## Abstract

**Background/Objectives:** To develop and validate a preoperative nomogram incorporating clinical and ultrasonographic features for the non-invasive differentiation of invasive ductal carcinoma (IDC) and invasive lobular carcinoma (ILC) of the breast. **Methods:** Preoperative clinical information and ultrasonographic features of patients with pathologically confirmed IDC and ILC were retrospectively collected. A total of 803 patients (600 with IDC and 203 with ILC) were enrolled and randomly allocated to training and validation sets in an 8:2 ratio. Univariate and multivariate logistic regression analyses were performed to identify independent predictors for differentiation. These predictors were subsequently incorporated into a nomogram and a corresponding weight plot. Model discrimination was assessed using the area under the receiver operating characteristic curve (AUC), while calibration was evaluated using calibration curves. Clinical net benefit was determined through decision curve analysis (DCA). **Results:** Significant differences were noted between IDC and ILC in multiple clinical and ultrasonographic characteristics (*p* < 0.05). Multivariate logistic regression analysis in the training set identified lesion margin, shape, depth, menopausal status, palpability, lesion classification, and internal echo as independent predictors of ILC. Notably, our constructed nomogram exhibited favorable predictive performance, calibration, and clinical utility in both the training and validation sets. **Conclusions**: A nomogram incorporating seven independent predictors, namely lesion margin, shape, depth, menopausal status, palpability, lesion classification, and internal echo, was developed and validated. This nomogram enables individualized and quantitative prediction of the preoperative probability of ILC and may serve as a non-invasive adjunct to support surgical decision-making.

## 1. Introduction

As one of the most prevalent malignancies among women worldwide, breast cancer has entered an era characterized by molecular subtyping and precision medicine [1]. Invasive ductal carcinoma (IDC) and invasive lobular carcinoma (ILC) represent the two predominant histological subtypes, accounting for approximately 70–80% and 5–15% of all breast cancer cases, respectively [2,3]. While therapeutic approaches for IDC and ILC largely overlap, accumulating evidence highlights the clinical relevance of preoperative differentiation in guiding surgical extent, sentinel lymph node biopsy strategies, selection of systemic therapy regimens, and long-term surveillance planning [4]. At present, histopathological examination remains the diagnostic gold standard, typically relying on postoperative specimens or preoperative core needle biopsy. However, the latter is subject to sampling limitations and potential procedure-related complications [5,6]. Consequently, non-invasive imaging-based diagnostic modalities have recently garnered extensive attention, with ultrasonography emerging as a cornerstone imaging modality for preoperative assessment owing to its widespread availability, real-time capabilities, and lack of ionizing radiation [7]. Previous studies have identified ultrasonographic morphological differences between IDC and ILC. For instance, ILC more frequently presents with irregular shapes, spiculated margins, and fewer calcifications, whereas IDC is more commonly associated with microcalcifications and clustered calcifications [8,9]. Nevertheless, current diagnostic practices predominantly rely on subjective interpretation of breast lesion characteristics by sonographers. The absence of systematic, quantitative, objective criteria has resulted in suboptimal diagnostic consistency and has limited the utility of ultrasonography as a reliable tool for clinical decision-making.

Within the current landscape of precision breast cancer management, a significant translational gap persists between research advancements and clinical practice. The clinical setting demands objective, efficient, and highly integrated decision-support tools. Although numerous advanced radiomics-based predictive models have demonstrated high discriminatory performance, their clinical adoption is constrained by complex workflows, limited interpretability, and challenges in integration into routine clinical workflows. Nomograms, however, transform multivariate logistic regression models into visual scoring tools that intuitively integrate clinical and imaging parameters, offering immediate, quantitative, individualized risk predictions while maintaining transparency and operational simplicity. These advantages confer outstanding clinical applicability and translational potential.

Thus, the present study aimed to develop and validate a nomogram that integrates routine preoperative clinical and ultrasonographic features to predict ILC. The innovation of this study lies in the development of a quantitative tool that combines objectivity, interpretability, and clinical utility, with the goal of addressing the subjectivity and uncertainty inherent in current differential diagnosis, assisting precise preoperative planning, and ultimately advancing individualized breast cancer management.

## 2. Materials and Methods

### 2.1. Study Population and Design

**Figure 1:**
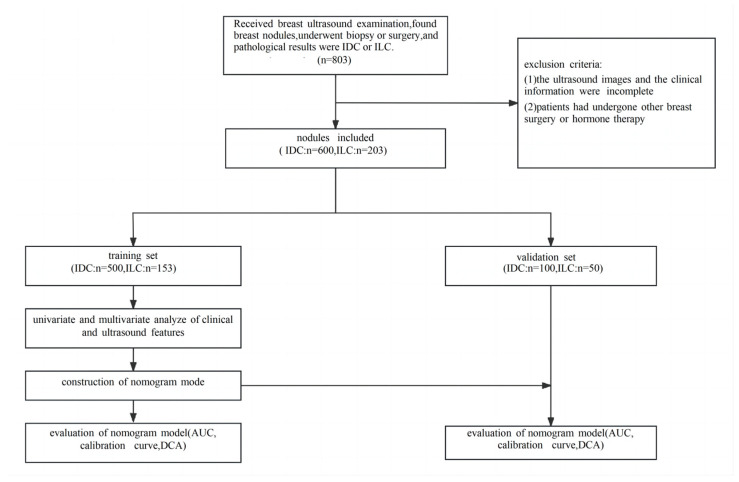
Study flowchart.

This retrospective study consecutively enrolled patients who underwent surgical interventions at the Department of Breast Surgery, the Second Qilu Hospital of Shandong University, between December 2020 and January 2024, with pathologically confirmed diagnoses of IDC or ILC. For patients with multiple lesions, the largest lesion was selected for analysis, yielding a final cohort of 803 patients (803 lesions). The inclusion criteria were as follows: (1) availability of complete clinical data and a pathologically confirmed diagnosis of ILC or IDC; (2) availability of complete ultrasound images obtained prior to biopsy or other interventional procedures. The exclusion criteria were as follows: (1) incomplete clinical information or ultrasonographic images; (2) history of prior surgery or hormonal therapy before presentation. Clinical data were retrieved from the inpatient electronic medical record system, while ultrasonographic images were acquired from the ultrasound workstation PACS system. Patients were randomly allocated to training and validation sets in an 8:2 ratio. This study was approved by the Ethics Review Committee of the Second Qilu Hospital of Shandong University (Ethics Number: KYLL202604627). Informed consent was obtained from all subjects involved in the study. The study flowchart is illustrated in Figure 1.

### 2.2. Instrumentation and Methods

Ultrasound examinations were performed using LOGIQ Fortis (GE Healthcare, Chicago, IL, USA), Resona 7S, and Eagus R9S (Mindray, Shenzhen, China) ultrasound systems equipped with linear array transducers (frequency range: 9–18 MHz). Patients were examined in the supine position, with both breasts and axillae scanned in transverse and longitudinal planes. Two-dimensional ultrasonographic features of each lesion were assessed and recorded according to the BI-RADS lexicon, including lesion length, width, depth, lesion classification, ductal abnormality status, echogenicity, internal echo, shape, echogenic halo, margin, posterior acoustic features, calcifications, aspect ratio, CDFI flow grade, and axillary lymph node metastasis. Blood flow signals were graded according to the Adler semi-quantitative method [10]: Grade 0: absence of intralesional blood flow; Grade I: low vascularity, with 1–2 punctate or short rod-like flow signals; Grade II: moderate vascularity, with one long vessel or 3–4 punctate vessels; and Grade III: high vascularity, with two long vessels or five or more punctate vessels. All ultrasound images were stored in the departmental PACS system. Two senior sonographers with over 10 years of experience independently analyzed ultrasonographic features. To evaluate inter-observer reliability, 60 images were randomly selected and independently reviewed by two senior sonographers (Expert A and Expert B). Agreement was assessed using Cohen’s kappa coefficient, with values > 0.75 reflecting good agreement. In cases of disagreement, consensus was reached through discussion and confirmed by a more senior physician.

Clinical data, including age, medical history (time from symptom onset to presentation), menopausal status, clinical symptoms, lesion multiplicity, and palpability, were collected from the inpatient electronic medical record system.

### 2.3. Statistical Analysis

Statistical analyses were performed using R software (version 4.3.3). Continuous variables conforming to a normal distribution were expressed as mean ± standard deviation, whereas non-normally distributed variables were reported as medians. For univariate analysis, normally distributed continuous variables were compared using independent samples *t*-tests, whereas non-normally distributed variables were analyzed using non-parametric tests (Mann–Whitney U test). Categorical variables were compared using Pearson’s chi-square test. Multivariate analysis was conducted using binary logistic regression, with *p* < 0.05 considered statistically significant. Receiver operating characteristic (ROC) curves were plotted, and the area under the curve (AUC) with 95% confidence intervals (CI) was used to evaluate model predictive performance. Calibration curves were constructed to assess model calibration. Decision curve analysis (DCA) was performed to evaluate clinical benefit. To assess correlations among ultrasound measurements (depth, length, width, and volume) and avoid multicollinearity, Pearson correlation analysis and collinearity diagnostics were performed. Multivariable binary logistic regression was subsequently conducted, with restricted cubic splines (RCS) incorporated to explore potential nonlinear relationships between continuous variables and the probability of ILC. Model calibration was evaluated using the Hosmer–Lemeshow test, and internal validation was carried out via 1000 bootstrap resamples. Agreement between predicted and observed probabilities was presented using calibration plots.

## 3. Results

### 3.1. Comparison of Clinical and Ultrasonographic Features Between Training and Validation Sets

A total of 803 patients meeting the inclusion and exclusion criteria were enrolled and randomly allocated to the training (n = 653) and validation (n = 150) sets in an 8:2 ratio. No significant differences were observed between the two sets in clinical or ultrasonographic features, except for pathological grouping (*p* = 0.012). Detailed results are presented in Table 1.

### 3.2. Comparison of Clinical and Ultrasonographic Features Between ILC and IDC Groups in the Training Set

Within the training set, 500 patients with IDC and 153 patients with ILC were included. Significant differences were observed between groups in menopausal status, lesion multiplicity, palpability, lesion classification, lesion length, width, depth, volume, L/D ratio, internal echo homogeneity, shape, and margin (*p* < 0.05). In contrast, no significant differences were noted in the remaining clinical or ultrasonographic features (*p* > 0.05). Detailed results are listed in Table 2.

### 3.3. Multivariate Logistic Regression Analysis and Nomogram Construction

**Figure 2:**
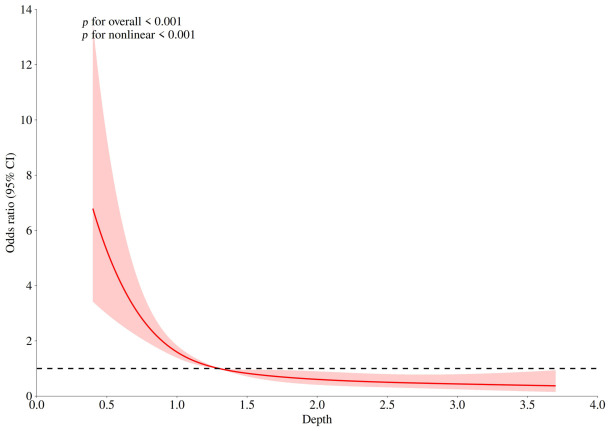
RCS curve. The red line represents RCS curve illustrating the relationship between depth and the risk of ILC. The red shaded area represents 95% CI. The black horizontal dashed line represents OR = 1.

**Figure 3:**
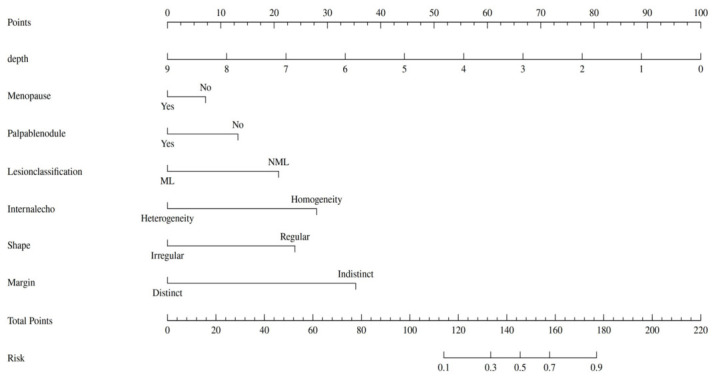
Nomogram for preoperative prediction of ILC.

**Figure 4:**
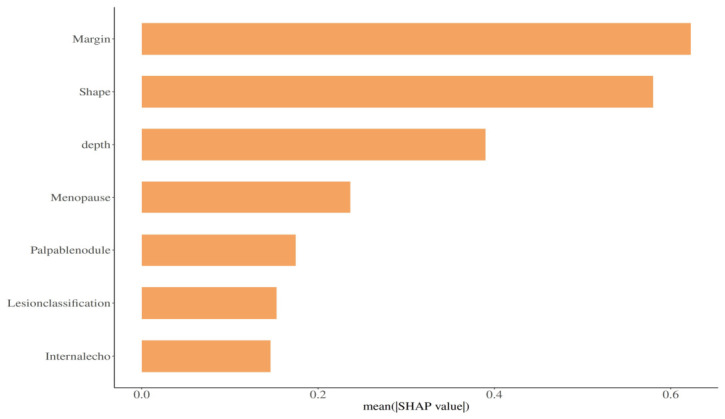
SHAP analysis of the model: feature importance bar plot.

**Figure 5:**
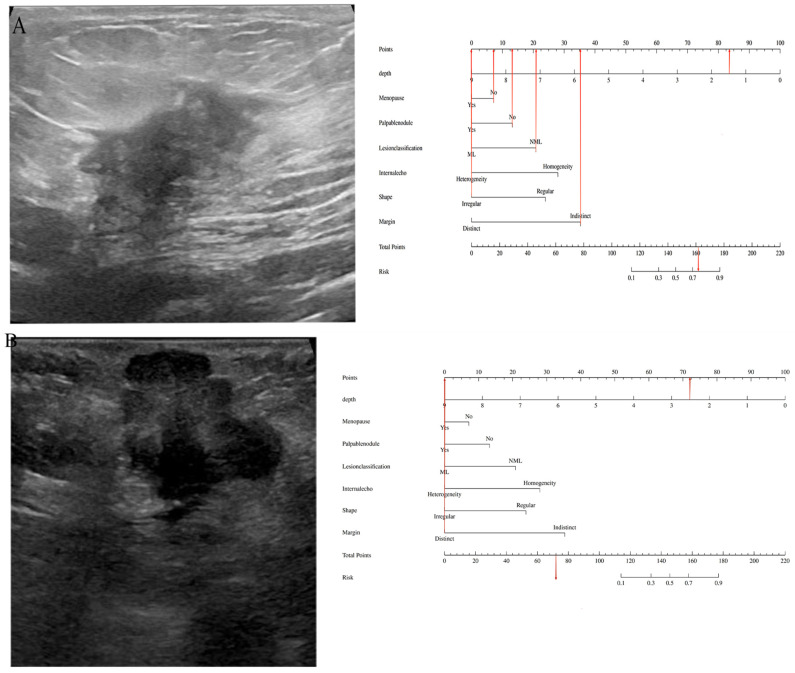
Ultrasonic cases and corresponding nomogram predictions. (**A**) A premenopausal female presented with a non-palpable breast lesion identified on ultrasound. Imaging revealed a non-mass lesion (21.5 points) measuring 3.1 cm × 1.4 cm × 1.8 cm, with a depth of 14 mm (84 points). The lesion exhibited an irregular shape (0 points), indistinct margins (35.5 points), and heterogeneous internal echotexture (0 points). Combined with premenopausal status (7 points) and non-palpability (13 points), the nomogram-generated total score was 161, corresponding to a predicted probability of invasive lobular carcinoma > 0.7. Histopathological examination confirmed the diagnosis of invasive lobular carcinoma of the breast. (**B**) A postmenopausal woman presented with a palpable breast mass. Ultrasound demonstrated a mass-type lesion (0 points) measuring 3.7 cm × 2.4 cm × 2.0 cm, with a depth of 24 mm (72 points). The lesion was characterized by an irregular shape (0 points), well-defined margins (0 points), and heterogeneous internal echotexture (0 points). Application of the clinical-ultrasound nomogram yielded a total score of 72 points (primarily attributable to lesion depth), corresponding to a predicted probability of invasive lobular carcinoma < 0.1. Histopathological examination established the diagnosis of invasive ductal carcinoma.

Pearson correlation analysis unveiled that lesion depth was strongly correlated with length, width, and volume (all r > 0.70). Multicollinearity diagnostics revealed significant collinearity for length (VIF = 6.581) and width (VIF = 7.512), both exceeding the threshold of 5, whereas depth (VIF = 2.928) and volume (VIF = 2.485) exhibited no substantial collinearity. In the multivariable logistic regression model incorporating all variables significant in univariable analyses, only lesion depth remained independently significant (*p* < 0.05). Restricted cubic spline (RCS) analysis revealed a non-linear dose–response relationship between lesion depth and the risk of ILC, with a reference value of 1.3 (odds ratio [OR] = 1) (Figure 2). As the depth increased, the risk of ILC significantly decreased, with a cut-off value of 1.3 cm and stabilizing after 2.5 cm. Next, clinically and ultrasonographically significant variables identified in univariate analysis were incorporated into the multivariate logistic regression model. Lesion depth, menopausal status, palpability, lesion classification, internal echo homogeneity, shape, and margin emerged as independent predictors with statistical significance (*p* < 0.05). Detailed results are displayed in Table 3. Based on the logistic regression analysis, a nomogram was constructed to predict ILC (Figure 3). The model incorporated variables with differential weights, including lesion depth, menopausal status, palpable nodule, lesion classification, internal echo, shape, and margin. SHAP analysis demonstrated that margin, shape, and lesion depth were the top three predictive factors. (Figure 4). As anticipated, the nomogram enabled differentiation between ILC and IDC through integration of various clinical and ultrasonographic parameters (Figure 5A, ILC case; Figure 5B, IDC case, respectively).

### 3.4. Nomogram Evaluation

**Figure 6:**
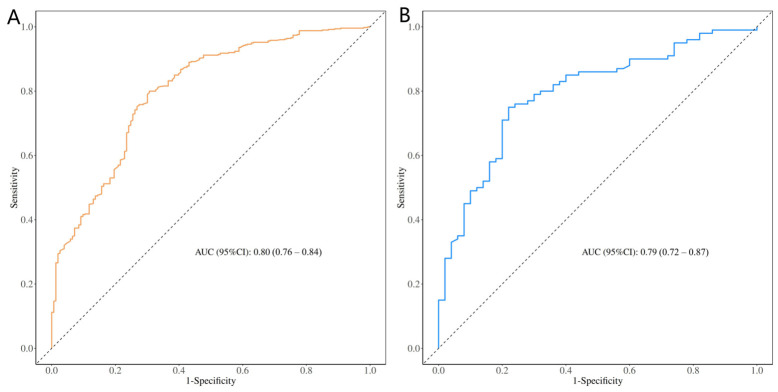
(**A**) ROC curve of the nomogram for predicting ILC in the training set. (**B**) ROC curve of the nomogram for predicting ILC in the validation set.

**Figure 7:**
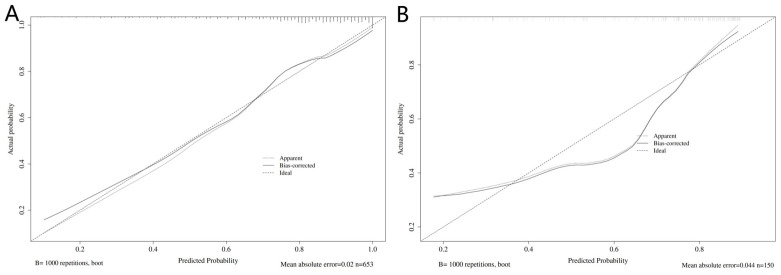
(**A**) Calibration curve of the nomogram for predicting ILC in the training set. (**B**) Calibration curve of the nomogram for predicting ILC in the validation set.

**Figure 8:**
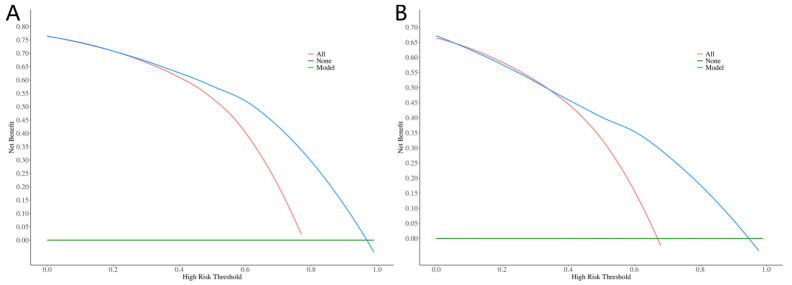
(**A**) Decision curve analysis of the nomogram for predicting ILC in the training set. (**B**) Decision curve analysis of the nomogram for predicting ILC in the validation set.

ROC curve analysis demonstrated that the nomogram achieved favorable diagnostic performance, with an AUC of 0.80 (95% CI: 0.76–0.84) in the training set (Figure 6A) and 0.79 (95% CI: 0.72–0.87) in the validation set (Figure 6B). Model calibration and potential overfitting were assessed using the Hosmer–Lemeshow goodness-of-fit test. In the training set, the χ^2^ value was 8.4699 (df = 8, *p* = 0.389). In comparison, the χ^2^ value was 4.3392 (df = 8, *p* = 0.825) in the validation set. Both *p*-values exceeded 0.05, indicating no significant discrepancy between predicted probabilities and observed outcomes with good calibration and no evidence of overfitting. At the same time, calibration plots demonstrated good agreement between predicted probabilities and actual outcomes in both training and validation sets (Figure 7A,B). Decision curve analysis revealed that the nomogram provided superior net clinical benefit across a wide range of threshold probabilities in both cohorts, substantially outperforming the extreme strategies of treating all or treating none. Overall, these findings reinforce the safety, reliability, and clinical utility of the constructed predictive model (Figure 8A,B).

## 4. Discussion

ILC and IDC represent the two predominant subtypes of breast cancer, and accurate preoperative differentiation between them is critical for optimizing treatment strategies [11]. The former originates from the terminal ductal-lobular unit, wherein loss of E-cadherin function leads to disrupted cell adhesion and a characteristic dyshesive growth pattern. This process manifests as an infiltrating carcinoma composed of non-cohesive cells arranged individually or in single-file linear patterns. The primary pathological feature involves the invasion of cancer cells through the basement membrane of terminal ducts or acini within breast lobules, followed by infiltrating into the lobular stroma. Disease progression proceeds from lobular atypia to carcinoma in situ and ultimately to invasive carcinoma [12,13]. IDC, in contrast, develops following disruption of the basement membrane by ductal carcinoma in situ in mammary ducts or acini, with subsequent invasion into the surrounding stroma and formation of invasive carcinoma [14]. Despite their distinct origins, ILC and IDC exhibit overlapping clinical and pathological features, contributing to diagnostic challenges.

Herein, a nomogram incorporating clinical and ultrasonographic features was developed and validated to predict ILC. Multivariate logistic regression analysis identified seven independent predictors, namely lesion depth, menopausal status, palpability, ultrasonographic lesion classification, internal echo, shape, and margin characteristics. This combination of predictors reflects the distinct biological behaviors of these two breast cancer subtypes as manifested through their clinical and imaging phenotypes.

In the present analysis, lesion margin characteristics, shape, and depth emerged as the three most heavily weighted predictors of ILC. Each predictor corresponds precisely to specific pathological mechanisms underlying ILC, collectively reflecting its fundamental nature of “dyshesive diffuse infiltration.” Indistinct lesion margins, representing the highest-weighted predictor positively associated with ILC diagnosis (OR = 11.7, *p* < 0.001), directly stem from the infiltrative growth pattern of the tumor. As cancer cells infiltrate along tissue planes in a non-cohesive manner, they neither generate sufficient mass-forming effect to establish a well-defined tumor-host interface nor elicit the prominent desmoplastic stromal reaction characteristic of IDC that would otherwise form a pseudocapsule [15]. Consequently, ultrasonography frequently displays ill-defined borders, frequently accompanied by characteristic elongated, linear “angular extensions” or spiculations that visually delineate the trajectory of cancer cell dissemination along anatomical planes. This finding not only aligns with the classical pathological features of ILC but also signifies that the constructed nomogram comprehensively captures and quantifies the critical imaging manifestations of ILC’s core biological behavior, substantially enhancing its clinical credibility and decision-support value.

In this study, “regular shape” emerged as the second most influential predictor associated with ILC (OR = 0.19, *p* < 0.001), revealing another crucial aspect of ILC growth dynamics. The compliant infiltrative growth of ILC lacks an expansive growth center, and the attenuated stromal response results in a macroscopic tumor contour that tends toward rounded configurations or gentle lobulated margins. This contrasts markedly with the stellate, irregular, or crab-like appearances frequently observed in IDC, which arise from heterogeneous growth patterns, necrosis, or intense desmoplastic reactions [6]. Notably, the combination of “regular shape” and “indistinct margins” in this study constitutes a characteristic ultrasonographic duality that accurately reflects the core biological behavior of ILC, namely, infiltrative yet non-expansive growth.

Lesion depth, defined as the anteroposterior dimension of the lesion on transverse ultrasound sections [16], ranked as the third most influential predictor in the analysis, with smaller lesion depth favoring ILC diagnosis (OR = 0.46, *p* < 0.001). The planar, sheet-like infiltrative pattern characteristic of ILC predisposes the tumor to preferential expansion along tissue planes parallel to the skin surface, significantly restricting anteroposterior growth and generally manifesting as flattened, discoid, or map-like hypoechoic areas on ultrasound [17]. On the other hand, the nodular expansive growth of IDC typically yields more spherical masses with relatively balanced enlargement across all dimensions. Thus, reduced lesion depth, particularly relative to greater longitudinal dimensions, is a crucial quantitative indicator of ILC’s planar growth pattern, providing essential complementary information about the three-dimensional spatial configuration.

Herein, premenopausal status, non-palpable lesions, non-mass lesion classification, and homogeneous internal echotexture were associated with an ILC diagnosis and served as central predictive factors. Of note, previous studies have reported a peak incidence of ILC between 51 and 61 years of age. However, in this study, no significant difference in mean age was noted between the ILC and IDC groups, with ILC patients being marginally younger (50.37 ± 10.74 years vs. 51.67 ± 10.69 years). Noteworthily, 88 patients (approximately 57.52%) with ILC were premenopausal, with a significant intergroup difference (*p* = 0.004), contrasting with the observations of prior studies [18,19,20]. This discrepancy can be ascribed to two factors: first, the trend toward younger age at breast cancer diagnosis may be more pronounced in ILC; the conclusion has been validated in several recent investigations [21,22]. Second, the relatively modest sample size of ILC in this study may have introduced statistical bias. It is worthwhile emphasizing that the predominance of non-mass lesions and non-palpable findings in ILC patients is consistent with its growth characteristics. ILC cells are relatively small, with uniform morphology and reduced cohesiveness, leading to diffuse, sheet-like infiltration within the fibrous stroma. This growth pattern fails to generate a well-defined mass, resulting in non-mass lesions with “flattened” configurations, thereby accounting for the non-palpable nature [23,24]. Interestingly, while homogeneous internal echotexture emerged as a predictive factor for ILC, it was relatively uncommon in both the ILC and IDC groups (3.92% vs. 1.00%). Heterogeneous echotexture typically results from tumor cell infiltration within connective tissue, forming cord-like or linear distributions that manifest as hyperechoic fibrous elements within hypoechoic lesions, or from calcification and necrosis. Herein, internal echo homogeneity was the lowest-weighted predictive factor.

Breast lesion calcifications are associated with focal necrosis, tissue dissolution, and subsequent calcium deposition. ILC cells diffusely infiltrate breast stromal tissue and adipose tissue with minimal or mild desmoplastic reaction and limited stromal destruction, resulting in fewer calcifications [8]. In this study, the proportion of lesions with calcifications in the ILC group was relatively high but lower than in the IDC group (48.37% vs. 53.40%), with no statistically significant intergroup difference (*p* = 0.330), diverging from the findings of previous reports [25]. The echogenic halo represents a common ultrasonographic sign in breast cancer, appearing as an abnormally hyperechoic structure at the interface between hypoechoic mass margins and adjacent glandular and adipose tissue. Characterized by ill-defined borders, frequent discontinuity, and marked thickness variability, it serves as a key prognostic indicator in breast cancer [26]. In this study, the proportion of lesions exhibiting an echogenic halo was lower in the ILC group than in the IDC group (14.38% vs. 20.40%), without a statistically significant intergroup difference (*p* = 0.097), consistent with findings reported by Yoon KH et al. [27].

Nevertheless, several limitations of this study warrant acknowledgment. To begin, the single-center, retrospective design and the exclusive inclusion of surgically treated patients may have introduced selection bias, thereby limiting generalizability. The relatively small sample size and lack of external validation further constrain the reliability of these findings. Second, a slight imbalance in the proportion of ILC cases between the training and validation cohorts could have influenced internal performance metrics. Third, certain findings were not in line with those of previous reports, and the underlying causes for these discrepancies remain to be elucidated. Fourth, the model achieved an AUC of approximately 0.80 in internal testing, which does not yet provide sufficient support for its clinical utility. Therefore, prospective multicenter studies with larger cohorts are warranted to provide more robust evidence and to evaluate potential clinical translation.

## 5. Conclusions

In summary, a clinical-ultrasound-based nomogram was developed and validated for the preoperative prediction of ILC. Seven independent predictors were identified, namely margin characteristics, shape, lesion depth, menopausal status, palpability, ultrasonographic lesion classification, and internal echo. Overall, the proposed model provides a quantitative tool for the non-invasive preoperative diagnosis of ILC, with potential utility in supporting individualized treatment decision-making.

## Figures and Tables

**Table 1:** Baseline characteristics of the study population.

Variables	Total (n = 803)	Training (n = 653)	Validation (n = 150)	T/χ^2^	*p*
Age (years)	51.16 ± 10.74	51.37 ± 10.70	50.24 ± 10.90	−1.16	0.247
Length (cm)	2.41 ± 1.41	2.42 ± 1.41	2.37 ± 1.45	−0.38	0.702
Width (cm)	1.93 ± 1.12	1.93 ± 1.12	1.90 ± 1.13	−0.32	0.747
Depth (cm)	1.50 ± 0.81	1.50 ± 0.81	1.49 ± 0.80	−0.17	0.865
Volume (cm^3^)	7.51 ± 21.76	7.47 ± 21.46	7.65 ± 23.09	0.09	0.928
L/D	1.66 ± 0.59	1.66 ± 0.60	1.62 ± 0.56	−0.79	0.428
Menopause, n (%)				1.68	0.195
Yes	413 (51.43)	343 (52.53)	70 (46.67)		
No	390 (48.57)	310 (47.47)	80 (53.33)		
Clinical symptoms, n (%)				0.07	0.798
No	145 (18.06)	119 (18.22)	26 (17.33)		
Yes	658 (81.94)	534 (81.78)	124 (82.67)		
Multiple lesions, n (%)				0.83	0.363
No	693 (86.30)	567 (86.83)	126 (84.00)		
Yes	110 (13.70)	86 (13.17)	24 (16.00)		
Palpable nodule, n (%)				1.42	0.233
No	95 (11.83)	73 (11.18)	22 (14.67)		
Yes	708 (88.17)	580 (88.82)	128 (85.33)		
Lesion classification, n (%)				1.32	0.250
NML	67 (8.34)	58 (8.88)	9 (6.00)		
ML	736 (91.66)	595 (91.12)	141 (94.00)		
Ductal abnormality, n (%)				1.02	0.312
No	757 (94.27)	613 (93.87)	144 (96.00)		
Yes	46 (5.73)	40 (6.13)	6 (4.00)		
Aspect ratio, n (%)				1.15	0.284
<1	636 (79.20)	522 (79.94)	114 (76.00)		
≥1	167 (20.80)	131 (20.06)	36 (24.00)		
Echotexture, n (%)				3.27	0.195
Hyperechoic	13 (1.62)	12 (1.84)	1 (0.67)		
Hypoechoic	764 (95.14)	617 (94.49)	147 (98.00)		
Mixed	26 (3.24)	24 (3.68)	2 (1.33)		
Internal echo, n (%)				0.00	1.000
Homogeneity	14 (1.74)	11 (1.68)	3 (2.00)		
Heterogeneity	789 (98.26)	642 (98.32)	147 (98.00)		
Shape, n (%)				0.25	0.615
Regular	82 (10.21)	65 (9.95)	17 (11.33)		
Irregular	721 (89.79)	588 (90.05)	133 (88.67)		
Margin, n (%)				0.03	0.852
Distinct	100 (12.45)	82 (12.56)	18 (12.00)		
Indistinct	703 (87.55)	571 (87.44)	132 (88.00)		
Echogenic halo, n (%)				3.05	0.081
No	641 (79.83)	529 (81.01)	112 (74.67)		
Yes	162 (20.17)	124 (18.99)	38 (25.33)		
Calcification, n (%)				0.81	0.667
Absent	389 (48.44)	312 (47.78)	77 (51.33)		
Macrocalcification	29 (3.61)	23 (3.52)	6 (4.00)		
Microcalcifications	385 (47.95)	318 (48.70)	67 (44.67)		
CDFI, n (%)				0.98	0.322
0-I	510 (63.51)	420 (64.32)	90 (60.00)		
II-III	293 (36.49)	233 (35.68)	60 (40.00)		
ALNM, n (%)				0.39	0.530
No	584 (72.73)	478 (73.20)	106 (70.67)		
Yes	219 (27.27)	175 (26.80)	44 (29.33)		
Pathology, n (%)				6.33	0.012
IDC	600 (74.72)	500 (76.57)	100 (66.67)		
ILC	203 (25.28)	153 (23.43)	50 (33.33)		

**Table 2:** Univariate analysis of clinical and ultrasonic features in the training set.

Variables	IDC (n = 500)	ILC (n = 153)	T/χ^2^	*p*
Age (years)	51.67 ± 10.69	50.37 ± 10.74	1.32	0.187
Length (cm)	2.52 ± 1.43	2.09 ± 1.29	3.31	<0.001
Width (cm)	2.02 ± 1.14	1.63 ± 0.99	3.88	<0.001
Depth (cm)	1.59 ± 0.84	1.19 ± 0.62	5.42	<0.001
Volume (cm^3^)	8.43 ± 23.80	4.35 ± 10.16	2.06	0.040
L/D	1.62 ± 0.54	1.80 ± 0.73	−2.83	0.005
Menopause, n (%)			8.08	0.004
Yes	278 (55.60)	65 (42.48)		
No	222 (44.40)	88 (57.52)		
Clinical symptoms, n (%)			2.90	0.088
No	84 (16.80)	35 (22.88)		
Yes	416 (83.20)	118 (77.12)		
Multiple lesions, n (%)			5.85	0.016
No	443 (88.60)	124 (81.05)		
Yes	57 (11.40)	29 (18.95)		
Palpable nodule, n (%)			24.54	<0.001
No	39 (7.80)	34 (22.22)		
Yes	461 (92.20)	119 (77.78)		
Lesion classification, n (%)			25.05	<0.001
NML	29 (5.80)	29 (18.95)		
ML	471 (94.20)	124 (81.05)		
Ductal abnormality, n (%)			0.84	0.361
No	467 (93.40)	146 (95.42)		
Yes	33 (6.60)	7 (4.58)		
Aspect ratio, n (%)			0.09	0.763
<1	401 (80.20)	121 (79.08)		
≥1	99 (19.80)	32 (20.92)		
Echotexture, n (%)			0.75	0.688
Hyperechoic	8 (1.60)	4 (2.61)		
Hypoechoic	473 (94.60)	144 (94.12)		
Mixed	19 (3.80)	5 (3.27)		
Internal echo, n (%)			4.40	0.036
Homogeneity	5 (1.00)	6 (3.92)		
Heterogeneity	495 (99.00)	147 (96.08)		
Shape, n (%)			15.53	<0.001
Regular	37 (7.40)	28 (18.30)		
Irregular	463 (92.60)	125 (81.70)		
Margin, n (%)			13.57	<0.001
Distinct	76 (15.20)	6 (3.92)		
Indistinct	424 (84.80)	147 (96.08)		
Echogenic halo, n (%)			2.76	0.097
No	398 (79.60)	131 (85.62)		
Yes	102 (20.40)	22 (14.38)		
Calcification, n (%)			2.22	0.330
Absent	233 (46.60)	79 (51.63)		
Macrocalcification	20 (4.00)	3 (1.96)		
Microcalcifications	247 (49.40)	71 (46.41)		
CDFI, n (%)			2.63	0.105
0-I	330 (66.00)	90 (58.82)		
II-III	170 (34.00)	63 (41.18)		
ALNM, n (%)			2.79	0.095
No	358 (71.60)	120 (78.43)		
Yes	142 (28.40)	33 (21.57)		

**Table 3:** Multivariate logistic regression analysis.

Variables	β	SE	Z	*p*	OR (95%CI)
Depth	−0.77	0.19	−4.16	<0.001	0.46 (0.32~0.66)
Menopause					
No					1.00 (Reference)
Yes	−0.50	0.21	−2.41	0.016	0.61 (0.40~0.91)
Palpable nodule					
No					1.00 (Reference)
Yes	−0.92	0.30	−3.06	0.002	0.40 (0.22~0.72)
Lesion classification					
NML					1.00 (Reference)
ML	−1.45	0.31	−4.67	<0.001	0.23 (0.13~0.43)
Internal echo					
Homogeneity					1.00 (Reference)
Heterogeneity	−1.95	0.75	−2.60	0.009	0.14 (0.03~0.62)
Shape					
Regular					1.00 (Reference)
Irregular	−1.67	0.33	−5.02	<0.001	0.19 (0.10~0.36)
Margin					
Distinct					1.00 (Reference)
Indistinct	2.46	0.54	4.55	<0.001	11.73 (4.06~33.86)

## Data Availability

The data presented in this study are available on request from the corresponding author due to restrictions privacy and legal.

## Abbreviations

The following abbreviations are used in this manuscript:

IDC	Invasive ductal carcinoma
ILC	Invasive lobular carcinoma
AUC	The area under the receiver operating characteristic curve
DCA	Decision curve analysis
BI-RADS	Breast Imaging Reporting and Data System
CDFI	Color Doppler Flow Imaging
ROC	Receiver operating characteristic curves
CI	Confidence intervals
L/D	Length/Depth
NML	Non-mass Lesions
ML	Mass lesions
